# Nontargeted Metabolomic Analysis of Plasma Metabolite Changes in Patients with Adolescent Idiopathic Scoliosis

**DOI:** 10.1155/2021/5537811

**Published:** 2021-05-25

**Authors:** Lige Xiao, Guanteng Yang, Hongqi Zhang, Jinyang Liu, Chaofeng Guo, Yang Sun

**Affiliations:** ^1^Department of Spine Surgery and Orthopaedics, Xiangya Hospital, Central South University, Changsha, China; ^2^National Clinical Research Center for Geriatric Disorders, Xiangya Hospital, Central South University, Changsha, China

## Abstract

**Objective:**

Adolescent idiopathic scoliosis (AIS) is a relatively common spinal rotation deformity, and the pathogenesis of AIS is accompanied by metabolic dysfunction and changes in biochemical factors. In this study, plasma metabolite changes in AIS patients were analyzed based on nontargeted metabolomics to provide new insights for clarifying functional metabolic abnormalities in AIS patients.

**Methods:**

Clinical indexes and blood samples were collected from 12 healthy subjects and 16 AIS patients. Metabolomics was used to analyze the changes in metabolites in plasma samples. The correlation between plasma metabolites and clinical indexes was analyzed by the Spearman rank correlation coefficient.

**Results:**

Analysis of clinical data showed that the body weight, body mass index (BMI), and bone mineral density (BMD) index of the AIS group significantly decreased, while the blood phosphorus and Cobb angles increased significantly. Metabolomic analysis showed significant changes in 72 differential metabolites in the plasma of the AIS group, mainly including organooxygen compounds, carboxylic acids and derivatives, fatty acyls, steroids and steroid derivatives, and keto acids and derivatives. The Kyoto Encyclopedia of Genes and Genomes (KEGG) pathway showed that arginine biosynthesis, D-glutamine and D-glutamate metabolism, alanine, aspartate and glutamate metabolism, and citrate cycle (TCA cycle) were significantly enriched in the AIS and healthy groups. Spearman rank correlation coefficient analysis showed that the plasma metabolites C00026 (oxoglutarate), C00062 (L-arginine, arginine), C01042 (N-acetylaspartate), and C00158 (citrate) were significantly correlated with clinical indexes in AIS patients. In the healthy group, the plasma metabolites C00122 (fumarate), C00025 (glutamate and L-glutamic acid) and C00149 (malate, L-malic acid) were significantly correlated with clinical indexes, while C00624 (N-acetylglutamate) was not significantly correlated with the clinical indexes.

**Conclusion:**

The occurrence of AIS led to changes in clinical indexes and plasma metabolites. Plasma biomarkers and functional metabolic pathways were correlated with clinical indexes, which might provide new insights for the diagnosis and treatment of AIS.

## 1. Introduction

Adolescent idiopathic scoliosis (AIS) is the most common spinal rotation deformity, affecting approximately 1-4% of adolescents worldwide [1]. Early diagnosis could increase the possibility of successful conservative treatment, thus reducing surgical intervention. AIS has a poor prognosis and a high mortality rate and can easily increase the risk of respiratory failure and cardiovascular disease [2]. The Abnormal neuromuscular system and calcium metabolism, as well as genetic and mechanical factors, might play a role in the pathogenesis of AIS. The physiological secondary effects of severe scoliosis were associated with restrictive lung disease, but the degree of deformity in most patients was not sufficient to affect their cardiopulmonary function. The clinical symptoms and pathological changes of AIS indicated that before abnormal growth of the spine, patients had metabolic dysfunction, and blood biochemical factors were involved in its pathogenesis, but the etiology of AIS was still unknown [3]. Metabolomics is a research method used to quantitatively analyze all metabolites in organisms and to search for the relative relationships between metabolites and physiological and pathological changes. It is an integral part of system biology [4–6]. Metabolomics had been widely used in the screening and diagnosis of disease biomarkers, which might be helpful for the early screening and treatment of AIS.

Ultra-performance liquid chromatography (UPLC) combined with quadrupole time-of-flight mass spectrometry (TOFMS) has been used to investigate the serum metabolic status between 30 AIS patients and 31 healthy controls. The metabolic profile of AIS patients usually deviated from that of healthy controls, and seven differential metabolites were identified as candidate diagnostic biomarkers, including PC (20 : 4), 2-hexanoylcarnitine, *β*-D-glucopyranosuronic acid, DG (38 : 9), MG (20 : 3), LysoPC (18 : 2), and LysoPC (16 : 0). These candidate metabolites indicated that lipid metabolism in AIS was blocked, including glycerophospholipid, glyceride, and fatty acid metabolism [7]. The increased expression of triglyceride lipase and hormone sensitive lipase in the adipose tissue further confirmed the increased lipid metabolism of AIS [8]. A domestic research team used metabolomics to analyze the changes in the gut microbiota metabolites of postmenopausal osteoporosis patients and identified the gut microbiota metabolites related to bone mineral density (BMD) and bone transformation markers, indicating that the changes in gut microbiota metabolites in postmenopausal osteoporosis patients were related to the decrease in BMD or bone metabolism indexes [9]. These studies demonstrated that serum differential metabolites found in AIS could be used as potential diagnostic biomarkers, and that lipid metabolism played a role in the pathogenesis of AIS.

In addition, most AIS patients have abnormal changes in estrogen and estrogen receptors, including increased serum estrogen concentrations, abnormal cellular responses to estrogen, older menarche age, and estrogen receptor gene polymorphisms, which are closely related to the susceptibility of AIS, the severity of the curve, and the progression of scoliosis [10, 11]. Plasma, the extracellular fluid of blood cells, is an important part of the internal environment of the body and plays an important role in communication between the internal and external environment of the body. In a recent study, Shen et al. found that the fecal microbial composition of AIS patients was different from that of healthy controls. Through proteomic analysis of the correlation between gut microbiota and plasma protein, it was confirmed that fecal Prevotella was positively correlated with host plasma FN1 and negatively correlated with host VDAC1 and AHNK [12]. The composition of plasma could be changed by the metabolic activity of the body and the external environment, but under normal circumstances, the body kept the composition of plasma relatively constant through various regulatory functions [13]. When the body is sick, the changes in some components in plasma might exceed the normal range. Therefore, the determination of plasma components might provide a basis for the diagnosis and treatment of AIS.

At present, there is no research on the plasma biomolecular metabolites of AIS patients. This study intends to use metabolomics to perform nontargeted broad screening of metabolites and analyze the differences in plasma metabolites between AIS patients and healthy subjects to elucidate the metabolic characteristics of AIS and provide a theoretical basis for the early screening of AIS.

## 2. Materials and Methods

### 2.1. Study Subjects

We studied subjects aged 12-18 years who were diagnosed with AIS from Hunan Province, China. Subjects completed a questionnaire about age, race, medication history, and medical history. The study excluded patients with other types of scoliosis caused by congenital or postural or neuromuscular factors and did not recruit subjects with acute infectious diseases, severe allergies, gastrointestinal diseases, and abnormal liver and kidney functions within the first month. None of the subjects underwent any surgery or rehabilitation. Height, weight, and body mass index (BMI) of all subjects were recorded.

### 2.2. Sample Preparation

The blood of all participants was centrifuged at 1150 g at 4°C for 10 minutes in a centrifuge tube coated with heparin. The supernatant was then aliquoted (100 *μ*L) into a labeled test tube and stored at -80°C before preparation for metabolomics analysis.

### 2.3. LC-MS/MS Analysis and Annotation

Samples (100 *μ*L) were mixed with 300 *μ*L of methanol and 20 *Μ*l of internal standard, vortexed for 30 s, extracted by ultrasonication in an ice water bath for 5 min, and stored at -20°C for 2 h. The mixture was centrifuged at 4°C (13,000 rpm, 15 min), and 200 *μ*L of supernatant was put into a 2 mL injection bottle for LC-MS/MS analysis. Baseline filtering, peak identification, integration, retention time correction, peak alignment, and normalization were performed on the original data obtained after mass spectrometry, and finally, a data matrix of retention time, mass/charge ratio, and peak intensity was obtained. Raw data were obtained for quality control, and bioinformatic analysis was performed.

MetaboAnalyst (https://www.metaboanalyst.ca/) was used for bioinformatic analysis. MetaboAnalyst is a comprehensive platform dedicated to metabolomic data analysis. There is a wide array of commonly used statistical and machine learning methods. In this study, the bioinformatic analyses, including univariate fold change, *t*-test, volcano plot, multivariate principal component analysis (PCA), partial least squares-discriminant analysis (PLS-DA), heatmap, and correlation analysis were applied. PCA is an unsupervised multidimensional statistical analysis method that can reflect the overall metabolic differences between samples in each group and the degree of variability between samples within the group [14]. PLS-DA is a multivariate statistical analysis method with supervised pattern recognition that groups multidimensional data according to the different factors that need to be searched before compression, so that the variables most relevant to the factors used for grouping could be found, and the influence of some other factors could be reduced [15]. The Kyoto Encyclopedia of Genes and Genomes (KEGG) pathway database was used to perform metabolic pathway enrichment analysis [16].

### 2.4. Correlation Analysis

The Spearman rank correlation coefficient and heatmap were generated to analyze the correlation between different metabolites and clinical indexes by Graph Prism 8.0 software.

### 2.5. Statistical Analysis

Statistical analysis of the data was performed using Graph Prism 8.0 and *R* software 3.1.0. The comparison between the two groups used Student's *t*-test or the Wilcoxon signed rank test, depending on whether the variable was normally distributed. *P* < 0.05 indicated that the difference was statistically significant.

## 3. Results

### 3.1. Characteristics of the Participants Involved in This Study

In this study, 12 healthy subjects and 16 AIS patients were collected, and their clinical data were statistically analyzed. Compared with the healthy group, the body weight and BMI of the AIS group significantly decreased, and the Cobb angle degree increased significantly. BMD measurements found that AIS patients had a significantly lower BMD index in the lumbar and femoral areas. Blood analysis showed that serum calcium and magnesium levels did not change significantly compared with the healthy group, while serum phosphorus levels increased significantly. The statistical analysis results of the clinical data are shown in Table 1. The above results indicate that the clinical indexes of AIS patients change to different degrees, which are closely related to the health of AIS patients.

### 3.2. Plasma Metabolism Profiles in AIS and Healthy Subjects

**Figure 1:**
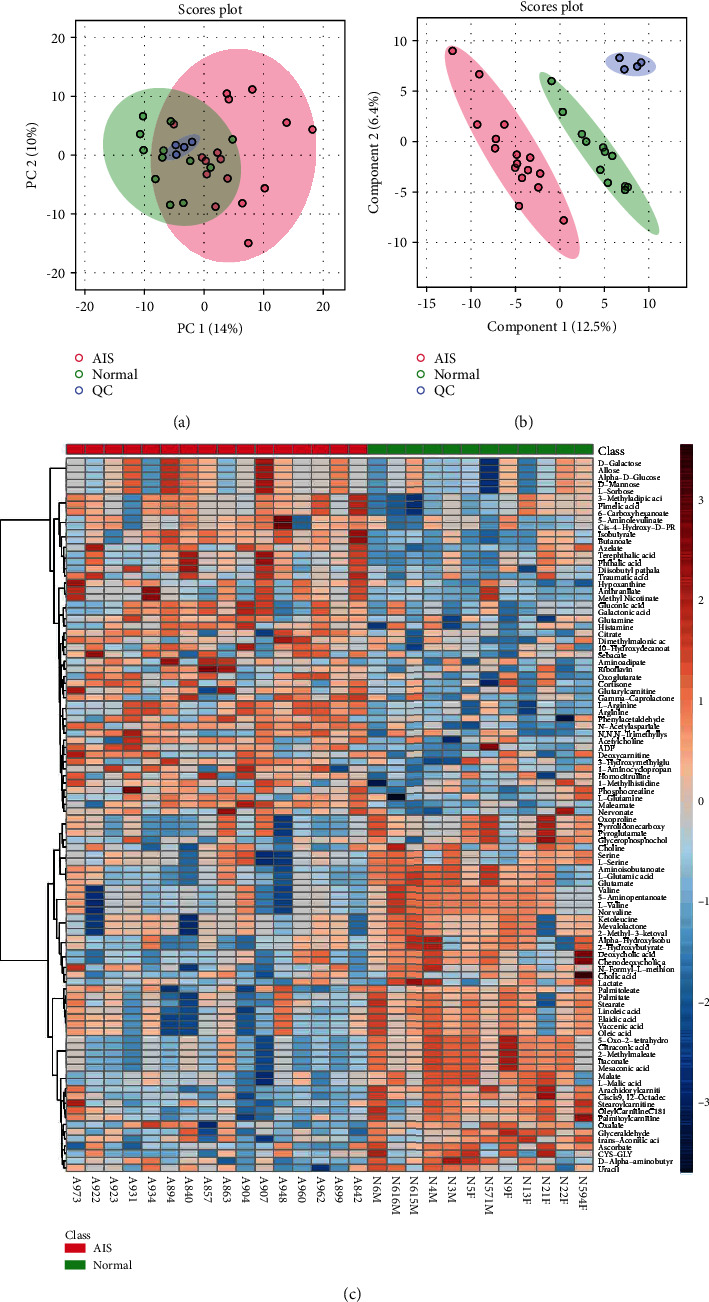
The analysis of plasma metabolite profiles. (a) PCA was used to analyze the degree of dispersion of samples in the quality control and healthy and AIS groups. (b) PLS-DA was used to analyze the degree of dispersion of samples in the quality control and healthy and AIS groups. (c) Heatmap shows the distribution of the top 100 metabolites among different samples.

To study the changes in metabolic phenotypes, plasma samples of healthy subjects and AIS patients were collected, and endogenous small molecules with relative molecular weights less than 1000 in plasma were widely screened by nontargeted metabolomics. Qualitative metabolites were based on public databases and self-built software databases. The normalized data matrix was used for multivariate statistical analysis, and PCA (Figure 1(a)) and partial least squares regression analysis (Figure 1(b)) were performed on all samples and quality control samples. PCA showed that the principal component 1 (PC1) index was 14%, and the PC2 index was 10%. PLS-DA analysis showed that the index of the variable factor was 12.5%. PCA and PLS-DA analysis showed uniform dispersion of quality control and grouped samples, which indicated that the stability and reliability of instrumental analysis were excellent and could be used for further analysis. A heatmap showed the top 100 metabolites obtained by nontargeted screening in the healthy group and AIS groups. It showed that the healthy group and AIS group were enriched with dominant metabolites (Figure 1(c)). These results suggested that the occurrence of AIS might lead to an abnormal plasma metabolome or AIS-related metabolic dysfunction.

### 3.3. Different Plasma Metabolism Profiles between AIS and Healthy Subjects

**Figure 2:**
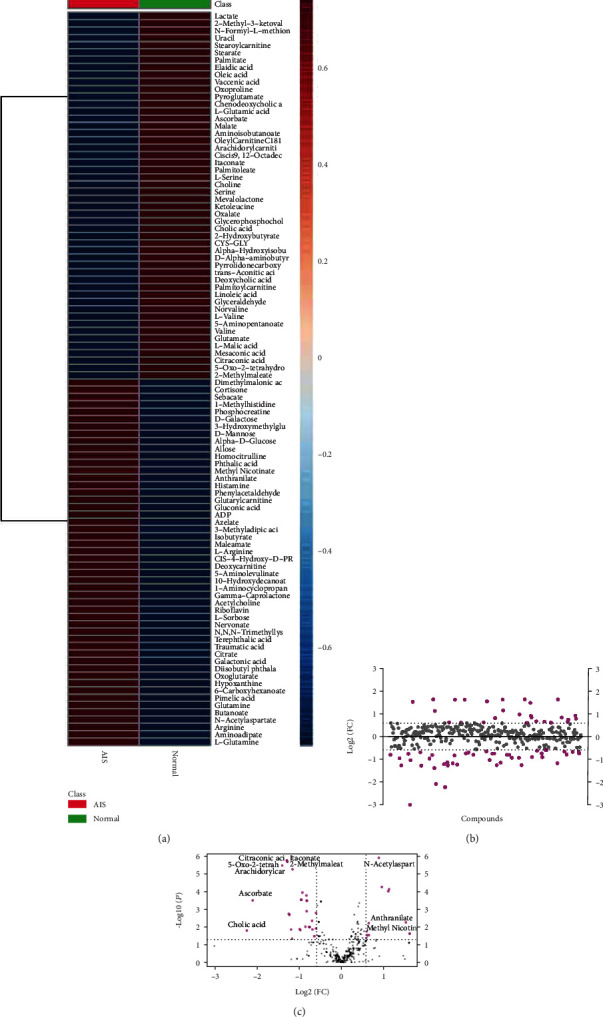
Analysis of the different metabolites. (a) Heatmap shows the difference in the top 50 metabolites between the healthy group and the AIS group. (b) Fold change diagram shows the fold change of different metabolites. (c) Volcano plot shows the changes in different metabolites.

To further analyze the differences in plasma metabolites between healthy subjects and AIS patients, we combined the VIP value of multivariate statistical analysis of OPLS-DA and the P value of the univariate statistical analysis *t*-test to screen the significantly different metabolites between different comparison groups. The threshold of significant difference was VIP ≥ 1 and *t*-test *P* < 0.05 [17]. After data normalization, cluster analysis and heatmaps were drawn to show the accumulation of different metabolites in the comparison groups [18]. A heatmap showed the top 50 metabolites significantly enriched in the AIS group and the healthy group (Figure 2(a)). The fold change diagram further showed the plasma fold changes of 72 different metabolites between the healthy group and the AIS group (Figure 2(b)). Volcano plots showed that 27 kinds of plasma differential metabolites were significantly increased, and 45 kinds were significantly decreased in the AIS group, and the absolute value of log2(FC) was ≥0.5869, *P* < 0.05 (Figure 2(c)).

Based on the Human Metabolome Database (https://hmdb.ca/), the differential metabolites in plasma were classified and summarized [19, 20]. Among them, the concentration of phenols anthranilate in the plasma metabolite class level of AIS patients increased. The concentration of methyl nicotinate in plasma pyridines and derivatives increased. The concentration of ribitol, L-arabitol, D-arabitol, D-xylitol, N-acetylgalactosamine, isopropanolamine, and trimethylamine N-oxide in plasma organooxygen compounds increased. The concentration of imidazopyrimidine hypoxanthine increased. The concentration of 4-acetamidobutanoate, N-acetylasparagine, L-arginine, arginine, N-acetylaspartate, homocitrulline, citrate, phosphocreatine, and dimethylmalonic acid in plasma carboxylic acids and derivatives increased. The plasma fatty acyl sebacate and nervonate concentrations increased. Plasma steroids and steroid derivatives taurodeoxycholic acid and taurocholic acid concentrations increased. The plasma keto acids and derivative oxoglutarate concentration increased. In addition, the unclassified maleamate, trigonelline, and monoethylmalonate concentrations increased. These results indicated that plasma metabolites were significantly altered in AIS patients, accompanied by the activation of metabolic pathways related to phenols, organic oxides, fatty acids, amino acids, and bile salts.

### 3.4. Metabolic Pathway Analysis between AIS and Healthy Subjects

**Figure 3:**
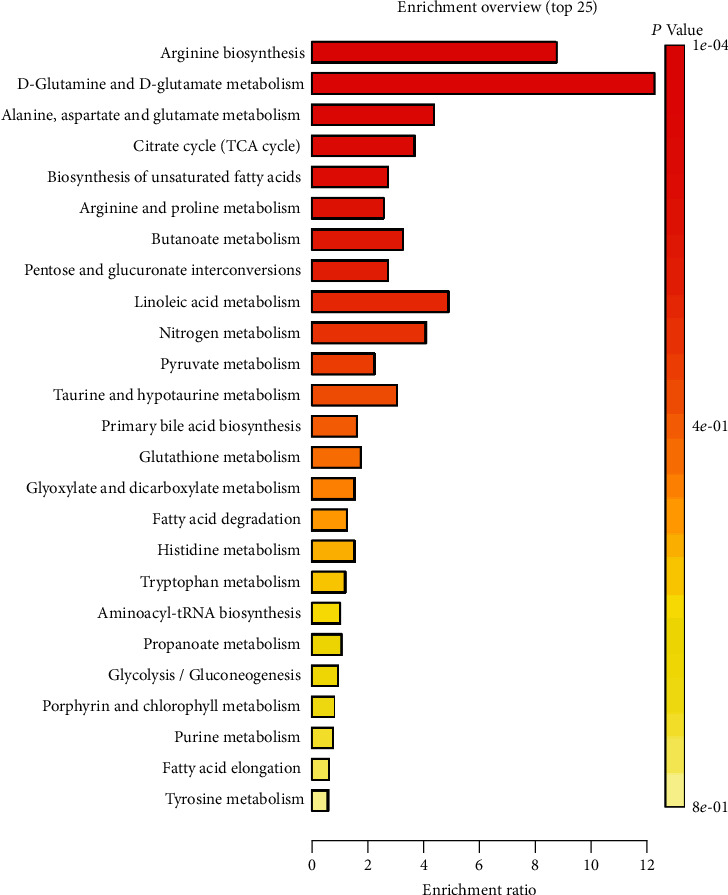
KEGG pathway analysis of metabolites.

To explore the functional changes caused by the changes in plasma metabolites in AIS patients, based on raw metabolomic data, signaling pathway function prediction analysis was carried out through the KEGG pathway database (https://www.kegg.jp/kegg/pathway.html). The KEGG pathway analysis showed the top 25 enriched signaling pathways in the AIS group and healthy group (Figure 3). There were significant changes in the L1 level metabolism functional pathway in the plasma of AIS patients. In the L2 level of amino acid metabolism, the L3 level of arginine biosynthesis was enriched in 14 plasma metabolites (*P* = 0.000144), of which C00026 (oxoglutarate) and C00062 (L-arginine, arginine) were significantly enriched in AIS patients, and C00624 (N-acetylglutamate), C00025 (glutamate, L-glutamic acid), and C00122 (fumarate) were significantly enriched in healthy subjects. At the L2 level of metabolism of other amino acids, the L3 level of D-glutamine and D-glutamate metabolism was enriched in 6 plasma metabolites (*P* = 0.000118), of which C00026 (oxoglutarate) was significantly enriched in AIS patients, and C00025 (glutamate, L-glutamic acid) was significantly enriched in healthy subjects. In the L2 level of amino acid metabolism, the L3 level of alanine, aspartate, and glutamate metabolism were enriched in 28 plasma metabolites (*P* = 0.000454), of which C00026 (oxoglutarate), C01042 (N-acetylaspartate), and C00158 (citrate) were significantly enriched in AIS patients, and C00025 (glutamate, L-glutamic acid) and C00122 (fumarate) were significantly enriched in healthy subjects. In the L2 level of carbohydrate metabolism, the L3 level of citrate cycle (TCA cycle) was enriched in 20 plasma metabolites (*P* = 0.0448), of which C00026 (oxoglutarate) and C00158 (citrate) were significantly enriched in AIS patients, and C00122 (fumarate) and C00149 (malate, L-malic acid) were significantly enriched in healthy subjects. The above results indicated that the significant accumulation and depletion of plasma metabolites in AIS patients were related to the functional metabolic preference for amino acid metabolism and energy cycling pathways.

### 3.5. Correlation between Plasma Metabolites and the Clinical Index

**Figure 4:**
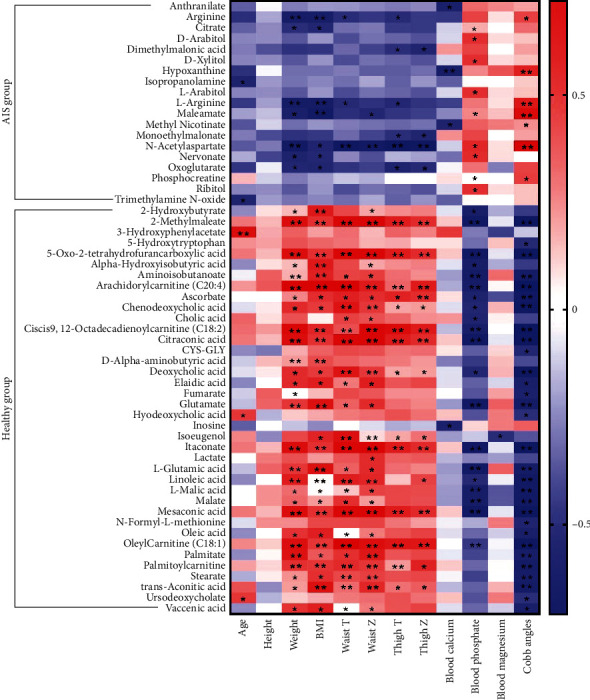
Correlation analysis of metabolites and clinical indexes.

Based on the clinical index characteristics of AIS patients and the plasma metabolomic analysis, we found that compared with healthy subjects, the clinical index of AIS patients showed serious adverse changes, and the types and functions of plasma metabolites of AIS patients were significantly changed. To further explore the changes between the plasma differential metabolites and the clinical index of AIS patients, the Spearman rank correlation coefficient was used to calculate the relationship between 72 plasma differential metabolites and the clinical index of AIS patients. Among them, 57 plasma differential metabolites were significantly correlated with the clinical index of patients (Figure 4). A heatmap showed that plasma metabolite C00026 (oxoglutarate) was negatively correlated with weight (*r* = −0.40, *P* = 0.031), BMI (*r* = −0.41, *P* = 0.029), and BMD (thigh *T*, *r* = −0.42, *P* = 0.024 and thigh *Z*, *r* = −0.38, *P* = 0.041) in AIS patients. C00062 (L-arginine) was significantly negatively correlated with weight (*r* = −0.51, *P* = 0.005), BMI (*r* = −0.50, *P* = 0.005), and BMD (waist *T*, *r* = −0.39, *P* = 0.038 and thigh *T*, *r* = −0.39, *P* = 0.036) but positively correlated with Cobb angles (*r* = 0.48, *P* = 0.009). C00062 (arginine) was significantly negatively correlated with weight (*r* = −0.49, *P* = 0.007), BMI (*r* = −0.49, *P* = 0.007), and BMD (waist *T*, *r* = −0.38, *P* = 0.043 and thigh *T*, *r* = −0.38, *P* = 0.042) and positively correlated with Cobb angles (*r* = 0.46, *P* = 0.012). C01042 (N-acetylaspartate) was significantly negatively correlated with weight (*r* = −0.53, *P* = 0.003), BMI (*r* = −0.41, *P* = 0.026), and BMD (waist *T*, *r* = −0.59, *P* < 0.001; waist *Z*, *r* = −0.53, *P* = 0.003; thigh *T*, *r* = −0.62, *P* < 0.001 and thigh *Z*, *r* = −0.59, *P* < 0.001), while positively correlated with blood phosphate (*r* = 0.43, *P* = 0.019) and Cobb angles (*r* = 0.71, *P* < 0.001). C00158 (citrate) was negatively correlated with weight (*r* = −0.37, *P* = 0.049) and BMI (*r* = −0.40, *P* = 0.032) and positively correlated with blood phosphate (*r* = 0.44, *P* = 0.018).

The plasma metabolite C00122 (fumarate) was significantly positively correlated with weight (*r* = 0.44, *P* = 0.017) and negatively correlated with Cobb angles (*r* = −0.45, *P* = 0.015) in healthy subjects. C00025 (glutamate and L-glutamic acid) was positively correlated with weight (*r* = 0.50, *P* = 0.005), BMI (*r* = 0.51, *P* = 0.005), and BMD (waist *T*, *r* = 0.38, *P* = 0.041 and waist *Z*, *r* = 0.40, *P* = 0.034), while negatively correlated with blood phosphate (*r* = −0.54, *P* = 0.002) and Cobb angles (*r* = −0.51, *P* = 0.005). C00149 (malate, L-malic acid) was positively correlated with weight (*r* = 0.43, *P* = 0.020), BMI (*r* = 0.41, *P* = 0.029), and BMD (waist *T*, *r* = 0.42, *P* = 0.024 and waist *Z*, *r* = 0.40, *P* = 0.030) and negatively correlated with blood phosphate (*r* = −0.49, *P* = 0.007) and Cobb angles (*r* = −0.54, *P* = 0.002). In addition, there was no significant correlation between clinical height index and the differentially enriched plasma metabolites in the AIS and healthy groups. There was no significant correlation between plasma metabolite C00624 (N-acetylglutamate) and the clinical index in healthy subjects. These results suggested that the significantly enriched and depleted metabolites in the plasma of AIS patients and clinical indexes might be used as AIS-related biomarkers for early screening.

## 4. Discussion

Early screening of AIS is still a major challenge in clinical diagnosis. In this study, metabolomics was used to deeply analyze the differences in plasma metabolites of AIS patients for the first time. Plasma organooxygen compounds, carboxylic acids, and derivatives were significantly enriched in AIS patients, and 57 metabolites were significantly correlated with clinical indexes. We found that plasma C00026 (oxoglutarate) was significantly enriched in AIS patients, and it was significantly negatively correlated with weight, BMI, and BMD (thigh T and thigh Z). Oxoglutarate, also known as *α*-ketoglutarate, AKG, or 2-oxoglutarate, was classified as a *γ*-keto acid or a *γ*-keto acid derivative. *α*-Ketoglutarate is a key molecule in the tricarboxylic acid (TCA) cycle and plays a fundamental role in determining the overall rate of this important metabolic process [21, 22]. In the TCA cycle, AKG is decarboxylated to succinyl coenzyme A and carbon dioxide by AKG dehydrogenase, which is the key control point of the TCA cycle. AKG is a nitrogen scavenger and a source of glutamic acid and glutamine, which can stimulate protein synthesis and inhibit protein degradation in muscles. AKG could reduce protein catabolism and increase protein synthesis, thereby enhancing the formation of the musculoskeletal tissue [21]. Interestingly, enteral feeding of AKG supplements could significantly increase circulating plasma levels of hormones such as insulin, growth hormone, and insulin-like growth factor 1 [21, 23]. Recent studies have shown that *α*-ketoglutarate promotes the differentiation of TH1 cells with the consumption of glutamine, which is beneficial to the differentiation of Tregs [24]. The enrichment of oxoglutarate in the plasma of AIS patients indicated increased demand for bone tissue formation, the disorder of hormone, and energy metabolism related to growth and metabolism in adolescents. Therefore, the regulation of plasma oxoglutarate might help promote the recovery of AIS.

C00062 (arginine) is an essential amino acid with physiological activity in the L-type strain. L-Arginine is an amino acid with multiple functions in the body that helps to process ammonia that is used to make compounds such as nitric oxide, creatine, L-glutamic acid, and L-proline and can be converted into glucose and glycogen when needed. At a high dose, L-arginine also stimulates the release of growth hormone and prolactin. Arginine is a known inducer of mTOR and is responsible for inducing protein synthesis through the mTOR pathway. The inhibition of mTOR by rapamycin partially reduces arginine-induced protein synthesis [25]. Catabolic diseases such as sepsis, injury, and cancer could lead to increased utilization of arginine, and the utilization of arginine might exceed normal body production, resulting in arginine consumption. Arginine also activated the AMPK pathway and stimulates skeletal muscle fatty acid oxidation and muscle glucose uptake, thereby increasing insulin secretion by pancreatic *β* cells [26]. This study found that plasma C00062 (L-arginine, arginine) was significantly enriched in AIS patients, and it was significantly negatively correlated with weight, BMI, and BMD (waist T and thigh T) and significantly positively correlated with Cobb angles. The massive enrichment of L-arginine and arginine in the plasma of AIS patients might be the metabolic dysfunction caused by AIS, and the reasonable regulation of plasma arginine metabolism might help the recovery and treatment of AIS.

C01042 (N-acetylaspartate) is a derivative of aspartic acid that is synthesized by aspartic acid and acetyl coenzyme A in neurons. Various functions of N-acetylaspartate are still under investigation, but the main proposed functions include acting as a neuroosmotic agent and participating in fluid balance in the brain, acting as a source of lipid acetate for the synthesis of myelin in oligodendrocytes, used as a precursor for the synthesis of the important dipeptide neurotransmitter N-acetylaspartyl glutamate (NAAG), and playing a potential role in the energy production of neuronal mitochondria [27, 28]. However, when N-acetylaspartate was present at a sufficiently high level, it could have a variety of adverse effects on many organ systems. Abnormally high levels of N-acetylaspartate in blood (organic acidemia), urine (organic aciduria), brain, and other tissues could cause general metabolic acidosis [29]. Adults with acidosis or acidemia are prone to headache, confusion, tiredness, tremor, lethargy, and flapping tremor. C01042 (N-acetylaspartate) was significantly enriched in the plasma of AIS patients, which might be related to the spinal injury caused by the AIS endangering spinal nervous system.

The plasma metabolites C00122 (fumarate), C00025 (glutamate and L-glutamic acid), and C00149 (malate, L-malic acid) were significantly enriched in healthy subjects and were closely correlated with clinical indexes. C00149 (malate, L-malic acid) is an organic dicarboxylic acid with a sour taste. Malic acid and fumarate are intermediate products of the TCA cycle [30, 31]. Under aerobic conditions, the oxidation of malic acid to oxaloacetic acid can provide a reduced mitochondrial equivalent through the redox shuttle between malic acid and aspartic acid. Under anaerobic conditions, the accumulation of excess reduction equivalents inhibits glycolysis, and the simultaneous reduction of malic acid to succinate and oxidation to oxaloacetic acid could remove the accumulated reduction equivalents, which leads to malic acid reversing the inhibition of hypoxia on glycolysis and energy production. C00122 (fumarate) is a dicarboxylic acid that has recently been identified as a metabolite or an endogenous carcinogenic metabolite. High levels of this organic acid can be found in tumors or biological fluids around tumors. Its carcinogenic effect seemed to be due to its ability to inhibit prolyl hydroxylase [32]. C00025 (glutamate and L-glutamic acid) is one of the 20 kinds of proteogenic amino acids. Glutamate is the most abundant fast excitatory neurotransmitter in the mammalian nervous system. Glutamate induced excitotoxicity was part of the ischemic cascade and is associated with stroke, amyotrophic lateral sclerosis, systemic lupus erythematosus, and Alzheimer's disease [33]. The depletion of the plasma metabolites C00122 (fumarate), C00025 (glutamate and L-glutamic acid), and C00149 (malate, L-malic acid) in AIS patients might be related to energy metabolism and spinal injury.

The study limitations mainly lie in the limited patient population covered by the analysis. The AIS patients selected in this study had mainly severe scoliosis with Cobb angles of 45.56 ± 12.78°. Meanwhile, since we studied the plasma metabolomics of multiple patients and did not specifically compare the gender and clinical classification of patients with King, Lenke, or PUMC (Peking Union Medical College) in AIS, our study may not fully represent the entire clinical spectrum of AIS patients. It has been reported that the Carter effect exists in AIS, and the incidence of AIS in women is 2-10 times higher than that in men [34–37]. In this study, we did not explore whether the gender or clinical classification affected the plasma metabolomics of AIS patients. Indepth follow-up studies will explore the above issue further.

In summary, this study used metabolomics to open the black box of plasma metabolomics in AIS patients. Based on the differential analysis of biomarkers, 72 small molecule metabolites were changed. Among them, the plasma metabolites C00026 (oxoglutarate), C00062 (L-arginine, arginine), C01042 (N-acetylaspartate), and C00158 (citrate) were significantly enriched in AIS patients. The plasma metabolites C00122 (fumarate), C00025 (glutamate and L-glutamic acid), C00149 (malate, L-malic acid), and C00624 (N-acetylglutamate) were significantly depleted. These eight AIS-related plasma biomarkers have significant effects on the functional metabolism of the following four signaling pathways: arginine biosynthesis, D-glutamine and D-glutamate metabolism, alanine, aspartate and glutamate metabolism, and citrate cycle (TCA cycle). Based on clinical index correlation analysis, it was found that these eight AIS-related plasma biomarkers were clearly correlated with clinical indexes, BMD, and Cobb angles or could be used as new targets for clinical screening and treatment of AIS.

## 5. Conclusion

The occurrence of AIS led to significant changes in clinical indexes, BMD, Cobb angles, and plasma metabolites. Changes in blood metabolism functional pathways were closely related to AIS-related plasma biomarkers. Plasma biomarkers and their functional metabolism might provide new insights for the screening and treatment of AIS.

## Figures and Tables

**Table 1:** Clinical information of the participants.

Participants	Control (*n* = 12)	AIS (*n* = 16)	*P* value
Age (years)	16.41 ± 3.20	15.12 ± 1.70	0.180
Gender (male/female)	6/6	6/10	0.508
Height (m)	1.70 ± 0.08	1.65 ± 0.08	0.169
Weight (kg)	73.18 ± 15.10	48.00 ± 10.44	<0.001
BMI (kg/m^2^)	25.36 ± 5.41	17.39 ± 2.73	<0.001
Cobb angles (°)		45.56 ± 12.78	<0.001
Bone density			
Waist *T*	−0.60 ± 1.23	−2.70 ± 1.03	<0.001
Waist *Z*	0.20 ± 1.04	−1.90 ± 1.13	<0.001
Thigh *T*	−0.64 ± 1.46	−2.75 ± 0.94	<0.001
Thigh *Z*	−0.18 ± 1.34	−2.15 ± 1.08	<0.001
Blood calcium (mmol/L)	2.32 ± 0.21	2.47 ± 0.35	0.213
Blood phosphate (mmol/L)	1.31 ± 0.14	1.58 ± 0.15	<0.001
Blood magnesium (mmol/L)	0.85 ± 0.07	0.95 ± 0.40	0.432

## Data Availability

The data used to support the findings of this study are available from the corresponding author upon request.
